# Intelligence and Sensory Sensitivity as Predictors of Emotion Recognition Ability

**DOI:** 10.3390/jintelligence5040035

**Published:** 2017-11-08

**Authors:** Katja Schlegel, Joëlle S. Witmer, Thomas H. Rammsayer

**Affiliations:** 1 Institute of Psychology, University of Bern, 3018 Bern, Switzerland; joelle.witmer@psy.unibe.ch (J.S.W.); thomas.rammsayer@psy.unibe.ch (T.H.R.); 2 Swiss Center for Affective Sciences, University of Geneva, 1202 Geneva, Switzerland

**Keywords:** emotion recognition ability, intelligence, sensory sensitivity, individual differences, emotional intelligence

## Abstract

The ability to recognize emotions from nonverbal cues (emotion recognition ability, ERA) is a core component of emotional intelligence, which has recently been conceptualized as a second-stratum factor of intelligence (MacCann et al., 2014). However, only few studies have empirically investigated the link between ERA, intelligence, and other mental abilities. The present study examined the associations between ERA, fluid intelligence, and sensory sensitivity in a sample of 214 participants. Results showed that both fluid intelligence and sensory sensitivity explained unique portions of variance in ERA. These findings suggest that future studies on ERA should include intelligence measures to assess the incremental validity of ERA above and beyond intelligence.

## 1. Introduction

Nonverbal emotional expressions serve important functions in interpersonal communication, such as signaling preferences, intentions, and relationships, and subsequently shaping the course and outcomes of social interactions [1]. Overall, healthy adults are relatively accurate at judging emotional expressions, even across different cultures [2]. However, there are also substantial individual differences in the ability to accurately perceive and interpret emotions from others’ faces, vocal tones, postures, and gestures, commonly labeled emotion recognition ability (ERA). This ability is typically measured with standardized performance-based tests in which participants are presented with pictures or recordings of emotional expressions, and are asked to choose which emotion label out of a list best describes each expression (for a brief overview, see [3]). 

A large body of research and several meta-analyses have demonstrated that higher ERA is associated with a wide range of psychosocial benefits, such as better mental health, social adjustment, relationship quality, and workplace performance (e.g., [4,5]). At the same time, deficits in ERA have been related to various mental disorders, such as schizophrenia [6], and to maladaptive traits such as trait anger, anxiety, and alexithymia [7]. The accurate recognition of emotions in others allow one to better understand and anticipate other people’s behavior, to adapt one’s own actions accordingly, and to smooth interactions [8]. As a consequence, better emotion recognition facilitates the attainment of one’s goals, ultimately resulting in better workplace outcomes (such as higher supervisor ratings), social adjustment, and relationship quality. Better social relationships predict better physical and mental health, for example, through more social support [9]. 

One potential mechanism through which ERA facilitates the understanding of another person’s emotions is emotional mimicry, which can be defined as sharing of an emotional nonverbal reaction of another person [10]. More accurate perception of others’ nonverbal cues, in particular of subtle and fast emotional changes, can result in stronger mimicry [11], and therefore in a better emotional understanding, which in turn promotes a shared emotional perspective and perceived similarity between the interaction partners, and helps to establish more successful and satisfying relationships [12]. Individuals with higher ERA might also generally be more interested and motivated with regard to understanding others [13], which is one prerequisite for emotional mimicry to occur [10]. 

While ERA has been studied for many decades, it has also been proposed as a central component in the more recent emotional intelligence construct. Emotional intelligence is conceptualized in two distinct ways; first, as a set of non-cognitive traits that are measured with self-report questionnaires (“trait EI”); and second, as a set of cognitive abilities that are measured with performance-based tests (“ability EI”; for an overview, see [14]). ERA fits in the ability EI approach which is represented by Mayer and Salovey’s [15] four-branch model, and defines EI as the ability to use emotional information efficiently to guide reasoning and behavior. In this model, ERA is part of the *emotion perception* branch, which is considered the most fundamental EI component, as it precedes the more complex branches of *emotion facilitation* (using emotions to facilitate reasoning), *emotional understanding* (knowledge about the qualities and determinants of emotions), and *emotion management* (the ability to regulate emotions in oneself and others). Trait EI models often include emotion perception as well, but define it as self-perceived clarity about one’s own and others’ feelings (e.g., [16]). Self-rated emotion perception skills are largely unrelated to performance-based ERA [17].

Although the term “emotional intelligence” implies a link to or similarity with traditional psychometric intelligence, there is surprisingly little research that examines the actual empirical relationship between ERA and intelligence, as well as other basic mental abilities. In their meta-analysis, Murphy and Hall [18] identified eight published sources with a total of 11 samples that correlated ERA with intelligence, the mean effect size being *r* = 0.22. However, most of these studies were conducted in the 1970s or earlier, long before the development of today’s standardized, state-of-the-art measures of ERA. In the EI field, more recent studies have examined the relationship between EI and traditional intelligence.

With respect to trait EI measures, there is now increasing consensus that they are unrelated to traditional psychometric intelligence, and might thus be more accurately labeled socio-emotional competency or socio-emotional effectiveness (e.g., [8,19,20]). With respect to ability EI, MacCann, Joseph, Newman, and Roberts [21] recently suggested that it represents an additional second-stratum factor of intelligence of similar standing as fluid intelligence. Nevertheless, based on 21 studies, Joseph and Newman [22] found a meta-analytic correlation of only *r* = 0.10 between the emotion perception branch and cognitive ability, which was the lowest correlation of all four ability EI branches. These results, however, must be interpreted with caution, as all studies in this meta-analysis used the same test, the Mayer-Salovey-Caruso Emotional Intelligence Test (MSCEIT; [23]). This test to date remains the only widely used performance-based measure that includes all four ability EI branches. Despite its widespread use, many researchers have criticized the psychometric properties of the MSCEIT, especially in relation to the rating scale response format and scoring based on consensus (e.g., [8,24,25]).

The emotion perception branch in the MSCEIT is measured through pictures of facial expressions as well as pictures of artwork, for which participants rate the extent to which different emotions are present. Responses are scored depending on the proportion of respondents in a normative general population or expert sample that chose each option (consensus scoring). That is, if 80% of the normative sample chose “not at all present” for one emotion, a respondent choosing the same option will receive a score of 0.80. This scoring format awards the highest scores to participants that agree with the majority of the population. As a consequence, MSCEIT scores are skewed towards the high end and do not discriminate among individuals in the higher ability range. Further, the test contains only four pictures of faces and five emotions, and the items featuring pictures of artwork are not consistent with the definition of ERA as emotion detection from nonverbal behavior. Recent ERA tests typically include several nonverbal modalities (face, voice, and/or body), more emotions (up to 14 in the case of the Geneva Emotion Recognition Test, GERT; [3]), and use a multiple choice format with an objectively defined correct answer (for a recent review of ERA measurement, see [26]. 

Taken together, past research suggests that ERA might be positively related to intelligence, but the results remain far from conclusive given the reliance on ERA measures that are either nonstandard, lack ecological validity by relying on few emotions and only still pictures of faces, and/or have other psychometric limitations. In addition, it is largely unknown how ERA relates to aspects of mental ability besides psychometric intelligence, such as individual differences in basic information processing. In what to our knowledge is the only available study, Castro and Boone [27] examined this question by correlating various spatiotemporal percepts with ERA measures. Results showed that sensitivity to rhythm, as well as sensitivity to angularity and other configural manipulations of lines (manipulations of the distance and angle of two lines), were positively related to accurate emotion recognition from facial and/or body movements. However, these authors did not examine ERA in vocal or multimodal emotional expressions. 

More research into different facets of elementary sensory information processing (such as auditory pitch discrimination and visual duration discrimination) would inform our understanding of the process of emotion recognition and the development of individual ERA.

### The Present Study

The aim of the present study was to extend previous findings on the association between ERA and general mental abilities. Specifically, this study assessed the relationship between multimodal ERA, fluid intelligence, and sensory sensitivity or sensory discrimination ability.

The ERA test used in this study, the GERT [3], measured ERA by presenting participants with short video clips with sound in which actors express 14 different emotions, and was developed and validated using Item Response Theory [3,7]. Given that in everyday life emotions are most often expressed dynamically and simultaneously in the face, voice, and body, the GERT responds to calls from several researchers for a broader, more comprehensive, and more ecologically valid assessment of ERA (e.g., [2,28]). In contrast, most other standard ERA tests focus on single modalities (typically static pictures of faces) and only few emotion categories, potentially restricting the generalizability of the respective findings [29].

In order to measure general mental ability, the present study used a standard test of fluid intelligence and two tasks measuring sensory sensitivity. Sensory sensitivity, or the ability to make fine discriminations in various sensory modalities, represents a basic component of information processing and shows substantial correlations with psychometric intelligence such that lower discrimination thresholds predict higher intelligence (e.g., [30,31,32,33]). This has been found in several modalities such as auditory (e.g., pitch discrimination; [32]), visual (e.g., color discrimination; [30]), and tactile (e.g., discriminate the form of stimuli applied to the skin; [34]). 

We propose that both higher sensory sensitivity (as indicated by lower sensory discrimination thresholds) and higher fluid intelligence will be correlated with higher ERA, as they are likely to be involved in the initial development of emotion perception skills (e.g., [1]). Higher sensory sensitivity might enhance the detection of subtle dynamic changes in facial muscle movements and vocal parameters, and thus facilitate the emotion recognition process at an early perceptual stage [35,36,37]. In contrast, higher fluid intelligence is likely to affect later stages of the emotion recognition process, including the retrieval of previous emotion knowledge, matching of this knowledge to the perceptual representation of the emotional expression, and assigning the correct verbal label to the expression [37]. Furthermore, higher fluid intelligence might facilitate the development of better emotion knowledge related to nonverbal cues and emotion vocabulary, and as a consequence, better ERA, throughout childhood and adulthood.

While women typically perform better than men in ERA tests (see meta-analyses by Hall [38], and Thompson & Voyer [39]), men were found to be better than women at pitch and loudness discrimination [40], and to reach higher scores on measures of fluid intelligence [41]. We therefore include gender as a covariate in the analyses. Finally, to ensure that the present results are specific to emotional abilities and do not generalize to self-perceptions of emotional competencies as conceptualized in trait EI models, this study also includes a trait EI questionnaire and examines its correlates with intelligence and sensory sensitivity. We expect that self-reported trait EI will be unrelated to actual performance on fluid intelligence, sensory sensitivity, and emotion recognition tasks. 

## 2. Method

### 2.1. Participants and Procedure

The sample consisted of 214 participants (108 women) ranging in age from 18 to 30 years (mean age ± standard deviation: 21.7 ± 2.5 years). Participants were recruited through flyers at the University of Bern and different vocational colleges, as well as through the participant pool of the psychology department, and received a compensation of CHF20. Eighty-one percent of the participants were university students and 19% were vocational school pupils or working persons of different professions without university entrance certification. All participants were naïve to the purpose of this study and had normal hearing and normal or corrected-to-normal vision. The study was approved by the ethics committee of the Faculty of Human Sciences, University of Bern. 

All instruments were administered in the laboratory during one testing session, except for the sensory sensitivity measures that were administered in a second session between one and two weeks after the first session. For 36 participants, all measures including sensory sensitivity were collected in one session for practical reasons. In addition to the instruments reported below, participants also completed experimental tasks involving psychophysiological measurements that are not part of the current analyses. Ten participants were excluded from the analysis because either (1) the CFT 20-R scores were more than two SD below the mean, completion time for the TEIQue was unusually long, and the experimenter had noted unusual behavior; or (2) their sensory threshold was more than three SD above the mean (i.e., they had a much lower sensory sensitivity) in one of the two tasks, suggesting that these participants did not follow the instructions properly. 

These participants were not part of the final *N* of 214. This sample size was adequate, as revealed by power analysis conducted with G*Power Version 3.1.9.2 [42]. This analysis indicated an *N* between 90 and 199 necessary to detect small-to-medium effect sizes (*f*^2^ of 0.04 to 0.09, corresponding to standardized regression coefficients of 0.20 to 0.30) for a power of .80 and an alpha level of 0.05. 

### 2.2. Measures

**Geneva Emotion Recognition Test short form** (GERT-S; [43]). The GERT-S is a computer-based test to measure ERA that consists of 42 brief video clips with sound (duration < 4 s) in which actors present 14 different emotions (six positive emotions: amusement, interest, joy, pride, relief, pleasure; seven negative emotions: anger, irritation, fear, anxiety, sadness, despair, disgust; and surprise). Each emotion is represented by three video clips, and all video clips are presented in pseudo-random order. The actors are shown from their upper torso upward so that postural/gestural and facial emotional cues are conveyed. In addition, in each clip vocal nonverbal cues are conveyed by a spoken sentence in a fantasy language without semantic meaning. After each clip, participants choose which of the 14 emotions best describes the emotion the actor intended to express. Responses were scored as correct (1) or incorrect (0), yielding a total score between 0 and 1. In addition, unbiased hit rates [43] were computed for the six positive emotions and the seven negative emotions, yielding a positive and a negative emotion score. Unbiased hit rates account for response biases towards certain response categories. In two validation studies, the GERT-S was shown to have high internal consistency ranging from *α* = 0.80 to *α* = 0.83, an essentially unidimensional structure, and substantial correlations with other test of emotion recognition abilities [43]. 

**Cattell’s Culture Fair Test, revised German version** (CFT 20-R; [44]). As a measure of psychometric intelligence, Part 1 of the German adaptation of Cattell’s Culture Fair Test was used. The CFT20-R Part 1 consists of four subtests. The first three subtests (Series, Classifications, and Matrices) are composed of 15 items and the fourth subtest (Topologies) of 11 items. Participants are required to infer complex relationships between elements or figures, such as completion of a series of figures (Series), identification of a deviant figure (Classification), completion of figure matrices (Matrices), and inferring topological relationships (Topologies). The aggregated score of the total number of correctly answered items in each of the four subtests was computed as a measure of psychometric intelligence. Internal consistency of the CFT 20-R Part 1 is *α* = 0.92 [44]. Previous studies on the validity of the CFT have shown that it primarily measures fluid intelligence, and represents an adequate marker of general mental ability (e.g., [44,45,46]). 

**Sensory discrimination**. To quantify individual sensory sensitivity, two discrimination tasks were used: a visual duration discrimination task and an auditory pitch discrimination task. Both tasks were fully computer-controlled and programmed in E-Prime experimental software (Psychology Software Tools, Inc., Sharpsburg, PA, USA). Each task consisted of 32 trials. On each trial, a standard and a comparison stimulus were presented successively, and participants were required to determine whether the first or the second stimulus was of longer duration, in case of the visual duration discrimination tasks, or of higher frequency, in case of the pitch discrimination task. The weighted up-down method [47], an adaptive psychophysical procedure, was employed to quantify individual discrimination performance. With this procedure, the difference between a constant standard stimulus and a variable comparison stimulus was varied from trial to trial depending on the participant’s previous response. After a correct response the difference was decreased (leading to a more difficult discrimination), after an incorrect response it was increased. 

For visual duration discrimination, stimuli were light flashes generated by a red LED positioned at eye level (diameter 0.38°, viewing distance 60 cm, luminance 68 cd/m^2^). A constant 100-ms standard interval and a variable comparison interval were presented with an interstimulus interval (ISI) of 900 ms. The initial duration of the comparison interval was 135 ms. During the first 6 trials, the difference between the standard and the comparison interval was decreased by 5 ms after a correct response and increased by 15 ms after an incorrect response. For the following trials, the step sizes were 3 ms and 9 ms, respectively. After each trial, participants had to decide whether the first or the second interval was longer by pressing one of two designated keys of the computer keyboard. In the auditory pitch-discrimination task, all stimuli were sine wave tones of the same duration (500 ms) and intensity (68 dB). Pitch of the constant standard tone was 440 Hz. The initial comparison tone had a frequency of 442 Hz. Step sizes according to the adaptive rule were 0.3 Hz and 0.9 Hz after a correct and incorrect response, respectively (0.5 Hz and 1.5 Hz for the first six trials). The ISI was 500 ms.

For both discrimination tasks, visual feedback was given immediately after the participant’s response (“+” for correct responses; “−” for incorrect responses) for 1500 ms. The next trial started 900 ms after the feedback. As a psychophysical measure of individual discrimination performance, the 75%-difference threshold was computed for both tasks. With this procedure, higher sensory sensitivity was indicated by smaller threshold values. The scores were z-standardized. The correlation (Spearman’s rho) between the two sensory discrimination tasks was *r* = 0.19 (*p* < 0.01). 

**Trait Emotional Intelligence Questionnaire** (TEIQue; [48]). The German adaptation of the TEIQue by [49] was used as a measure of global trait EI. The TEIQue is a paper and pencil test that consists of 153 items rated on a 7-point Likert scale ranging from 1 (absolutely disagree) to 7 (absolutely agree). It comprises 15 subscales yielding four factors (Emotionality, Self-Control, Sociability, Well-Being) and a global trait EI score. Given the 7-point Likert scale, an individual’s mean global TEIQue score can range from 1 to 7. As trait EI serves as a control variable in the present study, we only analyzed the global trait EI score. Internal consistency for the global TEIQue scale is high with Cronbach’s *α* = 0.96 [43]. In addition, numerous studies provided converging evidence for the predictive and construct validity of the TEIQue global score as an indicator of trait EI (e.g., [49,50,51]). 

## 3. Results

Table 1 shows the descriptive statistics for emotion recognition, cognitive ability, and trait EI measures, the results of *t*-tests comparing men and women, and correlations of all measures with gender. Women scored significantly higher on overall ERA, as measured by the GERT-S. Men scored significantly higher on psychometric fluid intelligence as measured by the CFT 20-R, and displayed a significantly lower visual sensory threshold, indicating better visual sensory sensitivity. There was no gender difference in self-reported trait EI and auditory pitch discrimination. 

Table 2 shows the correlations between all measures, including zero-order correlations (first part, upper right half), partial correlations controlling for gender (first part, lower left half), and separate correlations for men and women. Spearman’s rho was used because Shapiro-Wilk tests revealed that CFT 20-R and GERT-S scores were non-normally distributed. Partial correlations were computed because the mean values on most measures differed by gender. 

Results revealed a significant positive correlation (*r* = 0.26 *** controlling for gender) between GERT-S scores and the CFT 20-R, suggesting that higher psychometric intelligence is associated with better ERA. Results further showed significant negative associations between GERT-S scores and visual sensory discrimination threshold (*r* = −0.23 ** controlling for gender), suggesting that individuals with better ERA have higher visual sensitivity. The associations with auditory threshold pointed in the same direction, but were not statistically significant. 

The examination of the correlational patterns between GERT-S, gender, visual threshold, and the CFT 20-R suggest that gender acted as a suppressor variable. Gender (0 = female, 1 = male) was significantly correlated with GERT-S (*r* = −0.30 **), visual threshold (*r* = −0.40 **), and, to a lesser extent, the CFT 20-R (*r* = 0.14 *). GERT-S and visual threshold were only poorly correlated (*r* = −0.09), but when GERT-S was predicted from visual threshold with gender in the regression equation, the regression weight of visual threshold (*b* = −0.24 **) was substantially higher. A similar, but less pronounced pattern was found for the CFT 20-R as a predictor: The beta weight of the CFT 20-R predicting GERT-S with gender in the equation was 0.26 **, whereas the correlation between the CFT 20-R and ERA was only *r* = 0.21 **. 

In tendency, intelligence was more highly correlated with emotion recognition in women than in men (*r* = 0.30 *** vs. *r* = 0.18, n.s.), and visual sensory threshold was more highly correlated with emotion recognition in men than in women (*r* = −0.30 ** vs. *r* = −0.11, n.s.). As expected, the TEIQue was unrelated to the GERT-S, CFT 20-R, and sensory thresholds, suggesting that self-rated emotional competencies are largely independent of actual emotional and cognitive abilities. 

In order to further examine whether, firstly, intelligence and sensory discrimination uniquely explained variance in ERA and, secondly, these abilities were differentially related to ERA in men and women, we conducted a three-step multiple regression analysis with GERT-S scores as the dependent variable. In the first step, gender was the only predictor. In the second step, CFT 20-R scores and sensory thresholds were added to the model. In the third step, interaction terms of gender with CFT 20-R and sensory threshold scores were added. Results are shown in Table 3, and indicate that intelligence and visual sensory threshold, but not auditory sensory threshold, explained unique variance in ERA. However, the interaction terms with gender in the third step were not significant. It can therefore be concluded that intelligence and visual sensory threshold predict ERA similarly in men and women. 

In order to examine the unique and shared contributions of intelligence and visual sensory threshold to the prediction of ERA, a commonality analysis was conducted with GERT-S scores as the dependent variabe and CFT 20-R, visual sensory threshold, and gender as independent variables, following the procedures described by Nimon and colleagues [52] for R. The auditory threshold was not included in the analysis because it did not appear to be related to GERT-S scores. The results are presented in Table 4, and show that 23.32% of the variance explained in GERT-S scores (i.e., 0.04 of the total *R*^2^ of 0.17) can be uniquely attributed to the CFT 20-R and 14.26% (i.e., 0.02 of the total *R*^2^ of 0.17) can be uniquely attributed to the visual sensory threshold. Another 14.26% of the variance in the GERT-S can be accounted for by the common variance of the CFT 20-R and visual threshold scores. In addition, the commonality analysis yielded negative commonalities for all common effects involving gender (i.e., the last three common effects in Table 4). Negative commonality coefficients suggest the presence of suppression in that one variable confounds the predictive power of another (see [52,53]). Their magnitude indicates the power (variance explained) associated with including the confounding variable. In line with the above findings regarding gender as a suppressor variable, the negative commonalities indicate that the predictive power of visual threshold and, to a lesser extent, the CFT 20-R is bigger when gender is also included in the regression. Including gender in the regression model increased the unique contribution of visual threshold in predicting GERT-S scores by 13.96%, the unique effect of the CFT 20-R by 2.19%, and the common contribution of CFT 20-R and visual threshold by 10.19%. As the increase in unique explained variance due to inclusion of gender was much higher for visual threshold than for the CFT 20-R, it appears that gender acted as a suppressor variable particularly for visual threshold. The sum of the negative commonalities (2.19% + 13.96% + 10.19%) indicated that 26.34% of the regression effect was accounted for by the shared contributions involving gender. In other words, the explained variance in the regression model would have been 26.34% (about 0.04 of the *R*^2^ of 0.17) lower if the shared contribution of gender with the CFT 20-R and visual threshold had not been considered. A more detailed explanation of commonality analysis and the interpretation of negative commonality coefficients can be found in [52] and [53]. Overall, the results of the commonality analysis suggest that intelligence and visual threshold explain both unique and shared variance in ERA. When gender was accounted for, CFT 20-R and visual threshold explained a similar amount of unique variance (for CFT 20-R: 23.32% + 2.19% = 25.51%; for visual threshold: 14.26% + 13.96% = 28.22%). 

## 4. Discussion

The ability to recognize emotions from others’ nonverbal behavior is crucial to successful social interactions, and predicts better professional, academic, and interpersonal outcomes (e.g., [5]). It is also conceptualized as a core component of ability EI, which can be defined as the capacity to reason about emotions [54]. However, it remains largely unknown whether ERA is also related to general mental ability and information processing abilities. The present study, therefore, examined the associations between ERA, psychometric intelligence, and sensory sensitivity. To our knowledge, this study is the first one to use a multimodal ERA task simultaneously including visual and auditory (vocal) emotional expressions, as well as measures of both visual and auditory sensory sensitivity. 

Results showed that higher psychometric intelligence and higher visual sensory sensitivity (as indicated by lower thresholds in visual duration discrimination) were associated with better ERA, and that intelligence and visual sensory sensitivity explained unique and common variance in ERA. However, auditory sensory sensitivity (as indicated by lower threshold in pitch discrimination) was unrelated to ERA. These results are partly in line with the process model of emotion recognition proposed by Adolphs [37], in which a first stage of structural encoding of nonverbal features is followed by a second set of processes that links the perceptual properties of the expression to all pertinent knowledge components and ultimately results in the labeling of the perceived affective state. Higher sensitivity to visual information likely enhances the extraction and encoding of subtle changes in visual cues transmitted by the face and body in the early stages of the emotion recognition process, especially in dynamic emotion portrayals like the ones used in the present study. In later stages, higher fluid intelligence might facilitate the integration of the sensory information with existing representations of emotional expressions and the associated knowledge. At the final stage of the process, higher verbal ability (which is considered a main part of fluid intelligence [55,56]) might enable an individual to better differentiate the meaning of similar emotion words (e.g., the difference between anxiety and fear) and to understand the nonverbal signals associated with each emotion (e.g., [57]). The present results are also in line with Castro and Boone’s [27] findings that visual discrimination predicted accuracy in visual ERA tasks. 

The novel finding that auditory pitch discrimination did not predict ERA might suggest that the visual channel (i.e., facial cues, gestures, and body movements), on average, provides more information, or is more important, than the auditory channel (i.e., vocal or paralinguistic cues) when inferring an emotion from a video with sound. Previous studies found that for several emotions, such as disgust, contempt, pride, joy, and interest, recognition accuracy was much lower when the vocal channel was presented alone than when the visual (face and body) channel was presented alone [57]. These differences can be explained by the lack of specific or unique vocal cues for some emotions. For example, pride is often confused with irritation when presented only in the vocal channel as the vocal pattern is similar; the vocal expression of interest is not much different from the neutral voice; and disgust is not typically expressed in a sentence-like structure like in the GERT-S, but is characterized by a typical and unique facial expression that is easily recognized [58]. It could therefore be that a higher ability to detect subtle changes in pitch does not substantially contribute to a generally better ERA. Future studies should further disentangle the contribution of visual and auditory sensitivity by examining their associations with ERA in single modalities. It might be that auditory sensory sensitivity predicts ERA when measured in the vocal channel only. Future research is also needed to investigate how different facets of psychometric intelligence and other cognitive abilities, such as spatial ability, efficiency and speed of information processing, or attention, are functionally related to ERA. Such research could provide important insights into the mechanisms underlying individual differences in ERA and inform interventions to improve ERA. 

One noteworthy finding in the present study was that the effect sizes of the association between ERA and psychometric intelligence (*r* = 0.26 when controlling for gender) were substantially higher than the meta-analytic correlation of *r* = 0.10 found by Joseph and Newman [22] based on the MSCEIT emotion perception measure. Considering that the present study used a more comprehensive and ecologically valid ERA test than the MSCEIT, the effect sizes obtained here might be more veridical estimates of the strength of the ERA-intelligence association. In fact, the present results might still underestimate the actual relationships, because the present study was largely based on university students, who are relatively homogeneous in their cognitive abilities. 

This has major implications for future research examining the predictive validity of ERA. Given that psychometric intelligence is an important predictor of some of the same outcome variables as ERA, such as workplace performance [4,59], academic success [60,61], or income [62,63], it is important to test whether ERA has incremental validity over and above psychometric intelligence or whether ERA effects are already fully explained through the non-trivial correlation between ERA and intelligence. To date, however, most ERA studies have not controlled for intelligence. 

Another noteworthy finding was that higher self-ratings of EI (“trait EI”) were unrelated to ERA, intelligence, and sensory ability. These results support the idea that ability and trait EI are distinct constructs and that only ability EI (in this case, ERA as one basic ability EI component) displays a significant empirical association with psychometric intelligence (e.g., [8,14,21]). 

In the present sample, male and female participants differed in several ways. First, there was an overall mean difference in ERA favoring women and a mean difference in sensory sensitivity and psychometric intelligence favoring men. Similar differences have been found in other studies [39,40,41]. Although the multiple regression analysis did not show significant interactions of intelligence and sensory threshold with gender, the comparison of the correlation matrices for men and women could suggest that these predictors might not be equally important for both genders. Future research is needed to further study this question. In addition, the correlations between all performance-based variables for the full sample were larger when gender was controlled for. These findings imply that future research investigating associations between ERA and cognitive abilities should take gender into account as a potential moderator or suppressor variable. Due to the suppression effect of gender, associations between ERA and cognitive abilities might be missed or underestimated if gender is not included in the analyses. 

To conclude, the present study showed that psychometric intelligence and sensory sensitivity are important and independent predictors of ERA. These results also provide evidence for the validity of the GERT-S as a measure of ability EI and, at the same time, highlight the importance of controlling for cognitive ability in studies on ERA and ability EI more generally.

## Figures and Tables

**Table 1:** Descriptive statistics of cognitive abilities, emotion recognition, and trait emotional intelligence measures, and *t*-tests with effect size estimates comparing male and female participants.

	Total Sample (*n* = 214)		Females (*n* = 108)		Males (*n* = 106)		Gender Comparisons		Correlation with Gender (0 = Female, 1 = Male)
	*M*	*SD*	*M*	*SD*	*M*	*SD*	*t*(212)	*d*	*r*
CFT 20-R	45.30	4.96	44.6	5.0	46.1	4.8	−2.21 ***	−0.31	0.14 *
Auditory Threshold	0.00	1.00	0.08	0.92	−0.08	1.07	1.18	0.16	−0.11
Visual Threshold	0.00	1.00	0.40	0.99	−0.40	0.84	6.42 ***	0.87	−0.40 **
GERT-S Total Score	0.71	0.09	0.74	0.09	0.69	0.08	3.34 ****	0.59	−0.30 **
TEIQue	5.07	0.54	5.00	0.53	5.14	0.55	−1.99 *	−0.26	0.13

Note: GERT-S = Geneva Emotion Recognition Test short form, Hu = unbiased hit rate; CFT 20-R = Cattell’s Culture Fair Test—Revised German version, TEIQue = Trait Emotional Intelligence Questionnaire. Higher sensory threshold values represent lower sensory sensitivity. * *p* < 0.05; ** *p* < 0.01; *** *p* < 0.001 (two-tailed).

**Table 2:** Zero-order correlations and partial correlations (Spearman’s rho) between emotion recognition, psychometric intelligence, and trait emotional intelligence measures for the full sample (*n* = 214), and separately for males (*n* = 106) and females (*n* = 108).

	CFT 20-R	Visual Threshold	Auditory Threshold	GERT-S	TEIQue
*Full sample zero-order (lower left half) and partial correlations controlling for gender (upper right half)*					
(1) CFT 20-R		−0.29 ***	−0.18 **	0.26 ***	0.06
(2) Visual sensory threshold	−0.32 **		0.16 *	−0.23 **	0.08
(3) Auditory sensory threshold	−0.19 **	0.19 **		−0.11	0.00
(4) GERT-S	0.21 **	−0.09	−0.07		0.00
(5) TEIQue	0.08	0.02	−0.01	−0.04	
*Zero-order correlations for females (lower left half) and males (upper right half)*					
(1) CFT 20-R		−0.34 **	−0.16	0.18	−0.07
(2) Visual sensory threshold	−0.30 **		0.10	−0.30 **	0.03
(3) Auditory sensory threshold	−0.20 *	0.25 **		−0.13	0.09
(4) GERT-S	0.30 ***	−0.11	−0.03		−0.08
(5) TEIQue	0.18	0.14	−0.10	0.10	

Note: GERT-S = Geneva Emotion Recognition Test short form; CFT 20-R = Cattell’s Culture Fair Test—Revised German version, TEIQue = Trait Emotional Intelligence Questionnaire. Higher sensory threshold values represent lower sensory sensitivity. * *p* < 0.05; ** *p* < 0.01; *** *p* < 0.001.

**Table 3:** Multiple regressions predicting GERT-S scores from gender, visual and auditory sensory threshold, and psychometric intelligence.

	Step 1		Step 2		Step 3	
Independent Variables	*Beta*	*t*	*Beta*	*t*	*Beta*	*t*
(Constant)		42.97		11.40		2.70
Gender	−0.29 ***	−4.34	−0.39 ***	−5.65	0.36	0.59
Visual sensory threshold			−0.17 *	−2.39	0.00	−0.02
Auditory sensory threshold			−0.04	−0.56	0.06	0.29
CFT 20-R			0.21 **	3.06	0.44 *	2.13
Auditory Threshold * gender					−0.11	−0.51
Visual Threshold * gender					−0.18	−0.83
CFT 20-R * gender					−0.83	−1.22
Adjusted *R*^2^	0.08		0.16		0.15	

Note: Gender was coded 1 (women) and 2 (men). Higher sensory threshold values represent lower sensory sensitivity. * *p* < 0.05; ** *p* < 0.01; *** *p* < 0.001.

**Table 4:** Unique and common effects of intelligence and visual sensory threshold in predicting GERT-S scores.

	Commonality Coefficient	Percent Explained of *R*^2^
Unique to CFT 20-R	0.04	23.32
Unique to visual sensory threshold	0.02	14.26
Unique to gender	0.13	74.50
Common to CFT 20-R and visual sensory threshold	0.02	14.26
Common to CFT 20-R and gender	−0.00	−2.19
Common to visual sensory threshold and gender	−0.02	−13.96
Common to CFT 20-R, visual sensory threshold, and gender	−0.02	−10.19
Total *R^2^*	0.17	100.00

Note: GERT-S = Geneva Emotion Recognition Test short form; CFT 20-R = Cattell’s Culture Fair Test—Revised German version.

## References

[1] Knapp M.L., Hall J.A. (2009). Nonverbal Communication in Human Interaction.

[2] Elfenbein H.A., Ambady N. (2002). On the universality and cultural specificity of emotion recognition: A meta-analysis. Psychol. Bull..

[3] Schlegel K., Grandjean D., Scherer K.R. (2014). Introducing the Geneva emotion recognition test: An example of rasch-based test development. Psychol. Assess..

[4] Elfenbein H.A., Foo M.D., White J., Tan H.H., Aik V.C. (2007). Reading your counterpart: The benefit of emotion recognition accuracy for effectiveness in negotiation. J. Nonverbal Behav..

[5] Hall J.A., Andrzejewski S.A., Yopchick J.E. (2009). Psychosocial correlates of interpersonal sensitivity: A meta-analysis. J. Nonverbal Behav..

[6] Kohler C.G., Walker J.B., Martin E.A., Healey K.M., Moberg P.J. (2010). Facial emotion perception in schizophrenia: A meta-analytic review. Schizophr. Bull..

[7] Schlegel K., Fontaine J.R.J., Scherer K.R. (2017). The nomological network of emotion recognition ability. Eur. J. Psychol. Assess..

[8] Hampson E., van Anders S.M., Mullin L.I. (2017). A female advantage in the recognition of emotional facial expressions: Test of an evolutionary hypothesis. Evol. Hum. Behav..

[9] House J.S., Landis K.R., Umberson D. (1988). Social relationships and health. Science.

[10] Hess U., Fischer A. (2013). Emotional Mimicry as Social Regulation. Personal. Soc. Psychol. Rev..

[11] Hess U., Fischer A. (2014). Emotional mimicry: Why and when we mimic emotions. Soc. Personal. Psychol. Compass.

[12] Künecke J., Hildebrandt A., Recio G., Sommer W., Wilhelm O. (2014). Facial EMG Responses to Emotional Expressions Are Related to Emotion Perception Ability. PLoS ONE.

[13] Goh J.X., Schlegel K., Tignor S.M., Hall J.A. (2016). Who is interested in personality? The Interest in Personality Scale and its correlates. Personal. Individ. Differ..

[14] Roberts R.D., MacCann C., Matthews G., Zeidner M. (2010). Emotional intelligence: Toward a consensus of models and measures. Soc. Personal. Psychol. Compass.

[15] Mayer J.D., Salovey P., Salovey P., Sluyter D. (1997). What is emotional intelligence?. Emotional Development and Emotional Intelligence: Educational Implications.

[16] Petrides K.V., Furnham A. (2003). Trait emotional intelligence: Behavioural validation in two studies of emotion recognition and reactivity to mood induction. Eur. J. Personal..

[17] Riggio R.E., Riggio H.R., Hall J.A., Bernieri F.J. (2001). Self-report measurement of interpersonal sensitivity. Interpersonal Sensitivity: Theory and Measurement.

[18] Murphy N.A., Hall J.A. (2011). Intelligence and interpersonal sensitivity: A meta-analysis. Intelligence.

[19] Cherniss C. (2010). Emotional intelligence: Toward clarification of a concept. Ind. Organ. Psychol..

[20] Schlegel K., Grandjean D., Scherer K.R. (2013). Constructs of social and emotional effectiveness: Different labels, same content?. J. Res. Personal..

[21] MacCann C., Joseph D.L., Newman D.A., Roberts R.D. (2014). Emotional intelligence is a second-stratum factor of intelligence: Evidence from hierarchical and bifactor models. Emotion.

[22] Joseph D.L., Newman D.A. (2010). Emotional intelligence: An integrative meta-analysis and cascading model. J. Appl. Psychol..

[23] Mayer J.D., Salovey P., Caruso D.R., Sitarenios G. (2003). Measuring emotional intelligence with the MSCEIT v2.0. Emotion.

[24] Maul A. (2012). The validity of the mayer–salovey–caruso emotional intelligence test (MSCEIT) as a measure of emotional intelligence. Emot. Rev..

[25] Fiori M., Antonietti J.P., Mikolajczak M., Luminet O., Hansenne M., Rossier J. (2014). What is the ability emotional intelligence test (MSCEIT) good for? An evaluation using item response theory. PLoS ONE.

[26] Bänziger T., Hall J.A., Mast M.S., West T.V. (2016). Accuracy of judging emotions. The Social Psychology of Perceiving Others Accurately.

[27] Castro V.L., Boone R.T. (2015). Sensitivity to spatiotemporal percepts predicts the perception of emotion. J. Nonverbal Behav..

[28] Phillips L., Slessor G. (2011). Moving beyond basic emotions in aging research. J. Nonverbal Behav..

[29] Schlegel K., Boone R.T., Hall J.A. (2017). Individual differences in interpersonal accuracy: A multi-level meta-analysis to assess whether judging other people is one skill or many. J. Nonverbal Behav..

[30] Acton G.S., Schroeder D.H. (2001). Sensory discrimination as related to general intelligence. Intelligence.

[31] Deary I.J., Bell P.J., Bell A.J., Campbell M.L., Fazal N.D. (2004). Sensory discrimination and intelligence: Testing spearman’s other hypothesis. Am. J. Psychol..

[32] Helmbold N., Troche S., Rammsayer T. (2006). Temporal information processing and pitch discrimination as predictors of general intelligence. Can. J. Exp. Psychol..

[33] Troche S.J., Rammsayer T.H. (2009). Temporal and non-temporal sensory discrimination and their predictions of capacity- and speed-related aspects of psychometric intelligence. Personal. Individ. Differ..

[34] Stankov L., Seizova-Cajić T., Roberts R.D. (2001). Tactile and kinesthetic perceptual processes within the taxonomy of human cognitive abilities. Intelligence.

[35] Banse R., Scherer K.R. (1996). Acoustic profiles in vocal emotion expression. J. Personal. Soc. Psychol..

[36] Mortillaro M., Mehu M., Scherer K.R. (2011). Subtly different positive emotions can be distinguished by their facial expressions. Soc. Psychol. Personal. Sci..

[37] Adolphs R. (2002). Neural systems for recognizing emotion. Curr. Opin. Neurobiol..

[38] Hall J.A. (1978). Gender effects in decoding nonverbal cues. Psychol. Bull..

[39] Thompson A.E., Voyer D. (2014). Sex differences in the ability to recognise non-verbal displays of emotion: A meta-analysis. Cogn. Emot..

[40] Rammsayer T.H., Troche S.J. (2012). On sex-related differences in auditory and visual sensory functioning. Arch. Sex. Behav..

[41] Lynn R., Irwing P. (2004). Sex differences on the progressive matrices: A meta-analysis. Intelligence.

[42] Faul F., Erdfelder E., Lang A.G., Buchner A. (2007). G*power 3: A flexible statistical power analysis program for the social, behavioral, and biomedical sciences. Behav. Res. Methods.

[43] Schlegel K., Scherer K.R. (2016). Introducing a short version of the Geneva emotion recognition test (GERT-S): Psychometric properties and construct validation. Behav. Res. Methods.

[44] Weiss R.H. (2006). Grundintelligenztest Skala 2 (CFT 20-R) Mit Wortschatztest (WS) und Zahlenfolgentest (ZF)—Revision.

[45] Carroll J.B. (1993). Human Cognitive Abilities: A Survey of Factor-Analytic Studies.

[46] Cattell R.B. (1963). Theory of fluid and crystallized intelligence: A critical experiment. J. Educ. Psychol..

[47] Kaernbach C. (1991). Simple adaptive testing with the weighted up-down method. Percept. Psychophys..

[48] Petrides K.V., Stough C., Saklofske D.H., Parker J.D.A. (2009). Psychometric properties of the trait emotional intelligence questionnaire (TEIQue). Advances in the Measurement of Emotional Intelligence.

[49] Freudenthaler H.H., Neubauer A.C., Gabler P., Scherl W.G., Rindermann H. (2008). Testing and validating the trait emotional intelligence questionnaire (TEIQue) in a german-speaking sample. Personal. Individ. Differ..

[50] Mikolajczak M., Luminet O., Leroy C., Roy E. (2007). Psychometric properties of the trait emotional intelligence questionnaire: Factor structure, reliability, construct, and incremental validity in a french-speaking population. J. Personal. Assess..

[51] Petrides K.V., Pérez-González J.C., Furnham A. (2007). On the criterion and incremental validity of trait emotional intelligence. Cogn. Emot..

[52] Nimon K., Lewis M., Kane R., Haynes R.M. (2008). An R package to compute commonality coefficients in the multiple regression case: An introduction to the package and a practical example. Behav. Res. Methods.

[53] Nimon K. (2010). Regression commonality analysis: Demonstration of an SPSS solution. Mult. Linear Regres. Viewp..

[54] Mayer J.D., Salovey P., Caruso D.R. (2004). Emotional intelligence: Theory, findings, and implications. Psychol. Inq..

[55] Hunt E.B., Vernon P.A. (1987). The next word on verbal ability. Speed of Information-Processing and Intelligence.

[56] Mackintosh N.J. (2011). IQ and Human Intelligence.

[57] Rosip J.C., Hall J.A. (2004). Knowledge of nonverbal cues, gender, and nonverbal decoding accuracy. J. Nonverbal Behav..

[58] Bänziger T., Scherer K.R., Scherer K.R., Roesch E., Bänziger T. (2010). On the use of actor portrayals in research on emotional expression. Blueprint for Affective Computing: A Sourcebook.

[59] Schmidt F.L., Hunter J. (2004). General mental ability in the world of work: Occupational attainment and job performance. J. Personal. Soc. Psychol..

[60] Poropat A.E. (2009). A meta-analysis of the five-factor model of personality and academic performance. Psychol. Bull..

[61] Elfenbein H.A., Marsh A.A., Ambady N., Barrett L.F., Salovey P. (2002). Emotional intelligence and the recognition of emotion from facial expressions. The Wisdom in Feeling: Psychological Processes in Emotional Intelligence.

[62] Strenze T. (2007). Intelligence and socioeconomic success: A meta-analytic review of longitudinal research. Intelligence.

[63] Momm T., Blickle G., Liu Y., Wihler A., Kholin M., Menges J.I. (2015). It pays to have an eye for emotions: Emotion recognition ability indirectly predicts annual income. J. Organ. Behav..

